# Effect of the acromial inferolateral tilt on subacromial impingement syndrome: a retrospective magnetic resonance imaging assessment

**DOI:** 10.1590/0100-3984.2019.0127

**Published:** 2020

**Authors:** André Vaz, Camila Pietroski Reifegerste, Cesar Rodrigo Trippia, Lucas Savaris Linhares, Fábio Bordin Trindade, José Eduardo Thomaz

**Affiliations:** 1 Hospital Nossa Senhora das Graças, Curitiba, PR, Brazil; 2 Hospital São Vicente, Curitiba, PR, Brazil.

**Keywords:** Shoulder impingement syndrome, Inferolateral acromial tilt, Magnetic resonance imaging, Síndrome do impacto do ombro, Inclinação inferolateral do acrômio, Ressonância magnética

## Abstract

**Objective:**

To evaluate the effect of acromial inferolateral tilt on subacromial impingement syndrome.

**Materials and Methods:**

The acromial inferolateral tilt was retrospectively quantified by two researchers on 346 shoulder magnetic resonance images using the glenoacromial (between the inferior proximal acromial surface and the glenoidal face) and acromioclavicular (between the axis of the proximal acromion and distal clavicle) angles.

**Results:**

The glenoacromial angle was associated with subacromial impingement syndrome (*p* < 0.001) and complete supraspinatus tendon rupture (*p* < 0.001), and the acromioclavicular angle was associated with partial or complete supraspinatus tendon rupture (*p* = 0.003). The area under the receiver operating characteristic curve (AUC), best cut-off angle, and odds ratio (OR) of the glenoacromial angle for impingement syndrome were 0.579 (95% confidence interval [CI]: 0.508-0.649; *p* = 0.032), 72°, and 2.1 (95% CI: 1.136-4.053), respectively. For complete supraspinatus tendon rupture, the AUC, best cut-off angle, and OR of the glenoacromial angle were 0.731 (95% CI: 0.626-0.837; *p* = 0.001), 69°, and 8.496 (95% CI: 2.883-28.33), respectively. For partial or complete supraspinatus tendon rupture, the AUC, best cut-off angle and OR of the acromioclavicular angle were 0.617 (95% CI: 0.539-0.694; *p* = 0.002), 17°, and 3.288 (95% CI: 1.886-5.768), respectively. Interobserver agreement found for the glenoacromial and acromioclavicular angles were 0.737 (95% CI: 0.676-0.787; *p* < 0.001) and 0.507 (95% CI: 0.391-0.601; *p* = 0.001), respectively.

**Conclusion:**

Inferolateral acromial tilt may have some impact on subacromial impingement syndrome; however, the best quantification method identified (glenoacromial angle) showed a moderate interobserver agreement and a fair performance to assess the risk of complete supraspinatus tendon rupture.

## Abstract (pt)

**Objetivo:**

Avaliar a contribuição da inclinação inferolateral do acrômio na síndrome do impacto subacromial.

**Materiais e Métodos:**

A inclinação inferolateral do acrômio foi quantificada retrospectivamente por dois pesquisadores em 346 ressonâncias magnéticas de ombro por meio dos ângulos glenoacromial (entre a superfície inferior proximal do acrômio e a face glenoidal no plano coronal) e acromioclavicular (entre o eixo do acrômio proximal e o eixo da clavícula distal no plano coronal).

**Resultados:**

Houve associação entre ângulo glenoacromial e síndrome do impacto subacromial (*p* < 0,001) e ruptura completa do tendão supraespinal (*p* < 0,001). Ângulo acromioclavicular associou-se a ruptura parcial ou completa do tendão supraespinal (*p* = 0,003). A área sob a curva (*area under the curve* - AUC) característica de operação do receptor, o melhor ângulo de corte e a razão de chances (*odds ratio* - OR) do ângulo glenoacromial para a síndrome do impacto foram, respectivamente: 0,579 (intervalo de confiança [IC] 95%: 0,508-0,649; *p* = 0,032), 72° e 2,1 (IC 95%: 1,136-4,053). Para ruptura completa do tendão supraespinal, a AUC, o melhor ângulo de corte e a OR do ângulo glenoacromial foram, respectivamente: 0,731 (IC 95%: 0,626-0,837; *p* = 0.001), 69° e 8,496 (IC 95%: 2,883-28,33). Para ruptura parcial ou completa do tendão supraespinal, a AUC, o melhor ângulo de corte e a OR do ângulo acromioclavicular foram, respectivamente: 0,617 (IC 95%: 0,539-0,694; *p* = 0,002), 17° e 3,288 (IC 95%: 1,886-5,768). As concordâncias interobservador encontradas para os ângulos glenoacromial e acromioclavicular foram, respectivamente: 0,737 (IC 95%: 0,676-0,787; *p* < 0,001) e 0,507 (IC 95%: 0,391-0,601; *p* = 0,001).

**Conclusão:**

Inclinação inferolateral do acrômio pode determinar alguma influência sobre a síndrome do impacto subacromial, entretanto, o melhor método de quantificação identificado (o ângulo glenoacromial) apresentou moderada concordância interobservador e desempenho moderado para estratificar o risco de ruptura completa do tendão supraespinal.

## INTRODUCTION

Musculoskeletal disorders represent a significant part of general practice: 15% of primary health care appointments are related to this type of complaint^(1)^. Among them, shoulder pain is a major cause of morbidity, with a prevalence rate ranging from 6.7% to 21% in the population^(2)^. When compared to other orthopedic conditions, such as low back pain and osteoarthritis, shoulder pain has a large socioeconomic impact because it is associated with a lower work productivity and the need for longer sick leave^(3)^.

Shoulder impingement syndrome is the leading cause of chronic shoulder pain^(2,4-7)^. It is classified into external (or primary extrinsic and related to the coracoacromial arch) and internal impingement (or secondary extrinsic and related to glenohumeral or scapulothoracic instability)^(4)^. The etiology of external impingement is related to friction between the supraspinatus tendon and the inferior surface of the acromion, acromioclavicular joint, and coracoacromial ligament^(4)^. Risk factors of primary extrinsic impingement include accentuation of the acromial inferolateral tilt, Bigliani type III hooked acromion, low-lying acromion, os acromiale, inferiorly projecting acromioclavicular osteophytes, and bone deformities^(4,7-10)^.

Neer was one of the first to suggest that variation in acromial inclination could be a risk factor for impingement syndrome^(4)^. The lateral aspect of the acromion may incline inferiorly in the sagittal axis (condition called anteroinferior slope) or inferiorly in the coronal axis (condition called inferolateral tilt)^(7)^. The anteroinferior slope has been extensively studied; however, its effect on shoulder impingement syndrome remains controversial. While Bigliani et al.^(11)^, Morrison et al.^(12)^, Farley et al.^(13)^, and Epstein et al.^(14)^ found a statistically significant relationship between an anteriorly hooked acromion and shoulder impingement, Banas et al.^(15)^, Zuckerman et al.^(16)^, Chang et al.^(17)^, and Balke et al.^(18)^ could not reproduce such a relationship^(7,10)^. Furthermore, the impact of the acromial inferolateral tilt on subacromial impingement syndrome has been poorly studied.

The objective of the present study was to evaluate the effect of the acromial inferolateral tilt on subacromial impingement syndrome and to evaluate the interobserver variability of its quantification in shoulder magnetic resonance imaging (MRI).

## MATERIALS AND METHODS

This study complied with the Declaration of Helsinki and the Resolution 196/96 of the Brazilian National Council of Health regarding research involving humans. Because of the retrospective nature of this study, the need for informed consent was waived.

### Subject population

This retrospective study included all patients with shoulder pain who were ≥ 12 years old and underwent shoulder MRI at a private tertiary referral hospital between February 2016 and March 2019. The exclusion criteria were previous acromioplasty and other risk factors for subacromial impingement syndrome (including Bigliani type III hooked acromion, low-lying acromion, os acromiale, inferiorly projecting acromioclavicular osteophytes, and bone deformities, such as post-traumatic or related to Paget disease).

### Imaging parameters

All shoulder MRI scans were performed on a 1.5-T Advantage Excelart (Toshiba Corp., Tokyo, Japan) with a dedicated shoulder coil (4 Channel Array Adapter MJCC-167A, Toshiba) and with the patient in anatomical position. Coronal oblique proton density-weighted sequences were used for the measurements. The oblique coronal plane was obtained along the long axis of the supraspinatus tendon and perpendicular to the oblique sagittal plane. The proton density sequences were acquired with fat saturation using the following parameters: repetition time = 1890 ms, echo time = 30 ms, field of view = 19 cm, slice thickness = 3.5 mm, interslice gap = 0.3 mm, and matrix size = 272 × 272.

### Data collection and image analysis

**Figure 1:**
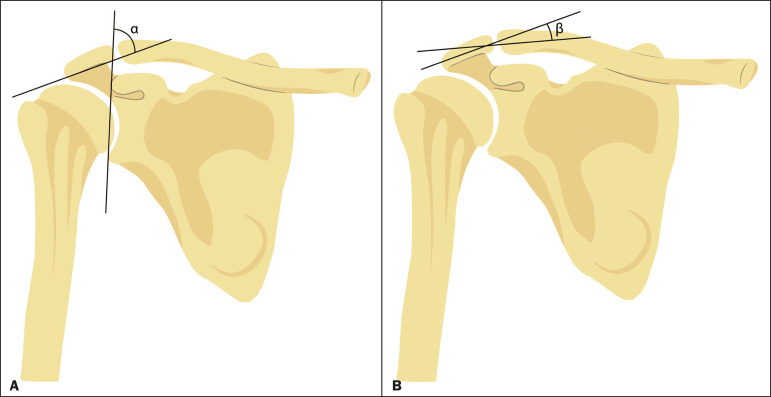
Methods of quantifying the inferolateral acromial tilt. A: The glenoacromial angle (α) is the angle between the glenoid face and the lower surface of the acromion. B: The acromioclavicular angle (β) is the angle between the axes of the proximal end of the acromion and distal end of the clavicle.

Information regarding age and sex was collected from each patient’s medical chart. All included cases were retrieved from the hospital’s picture archiving and communication system and reviewed between June and July 2019 without prior knowledge of patient treatment or outcome. Two researchers (observer A and observer B) independently quantified the inferolateral acromial tilt angle using two different methods: the glenoacromial angle described by Banas et al.^(15)^ and the acromioclavicular angle described by MacGillivray et al.^(19)^ (Figure 1). The Banas’ glenoacromial angle was defined as the angle between the inferior surface of the proximal end of the acromion and glenoid face on the oblique coronal plane images, just posterior to the acromioclavicular joint^(15)^. The MacGillivray’s acromioclavicular angle was defined as the angle between the axis of the midsubstance of the distal end of the clavicle and the axis of the midsubstance of the proximal end of the acromion on coronal oblique plane images, at the level of the acromioclavicular joint^(19)^.

**Figure 2:**
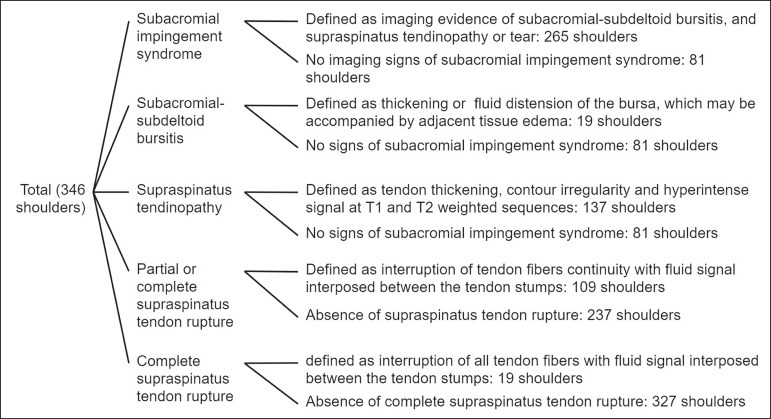
Flowchart demonstrating group division.

Because of the retrospective nature of the study, we did not have access to detailed patient clinical data (many were clinically evaluated at other health centers); therefore, data related to the outcomes were collected from the MRI reports by the authors. Subacromial impingement syndrome is clinically defined as shoulder pain when performing abduction with external rotation or flexion with internal rotation and is caused by subacromial-subdeltoid bursitis, tendinopathy, or supraspinatus tendon rupture, which are easily characterized on MRI^(4)^. Therefore, individuals with such alterations were classified as subacromial impingement syndrome despite its clinical definition. The findings of each report were classified as follows (Figure 2 contains a flowchart clarifying this classification): 1) absence of imaging-based evidence of subacromial impingement syndrome; 2) imaging-based evidence of subacromial impingement syndrome (defined as an imaging evidence of subacromial-subdeltoid bursitis, and supraspinatus tendinopathy or tendon rupture); 3) subacromial-subdeltoid bursitis (defined as a thickening or fluid distension of the bursa, which may be accompanied by adjacent tissue edema or enhancement); 4) supraspinatus tendinopathy (defined as tendon thickening, contour irregularity, and hyperintense signal on T1 and T2 weighted sequences); 5) partial or complete supraspinatus tendon rupture (partial or full-thickness tears, defined as an interruption of tendon fiber continuity with fluid signal interposed between the tendon stumps); and 6) complete supraspinatus tendon rupture (defined as an interruption of all tendon fibers with fluid signals interposed between the tendon stumps).

### Statistical analysis

The included data was entered into a SPSS version 23 (IBM Corp., Armonk, NY, USA) database. The Kolmogorov-Smirnov and Shapiro-Wilks tests were used to test the normality of the values. Univariate analysis was performed using non-parametric Mann-Whitney U test. Binary logistic regression was performed for multivariate analysis. Goodness of fit was evaluated by Nagelkerke’s R squared and Hosmer and Lemeshow test. *P*-values < 0.05 were considered statistically significant.

Receiver operating characteristic (ROC) curves were plotted to evaluate the accuracy, and the optimal angle criterion was calculated using Youden’s index in the statistically significant variables^(20)^. The performance of the diagnostic test was classified as fail (area under the curve [AUC] between 0.5 and 0.6), poor (AUC between 0.6 and 0.7), fair (AUC between 0.7 and 0.8), good (AUC between 0.8 and 0.9), and excellent (AUC between 0.9 and 1)^(21)^.

Interobserver variability was evaluated using intraclass correlation coefficient (ICC), and the agreement was classified as poor (ICC < 0.50), moderate (ICC between 0.50 and 0.75), good (ICC between 0.75 and 0.90), and excellent (ICC > 0.90)^(22)^.

## RESULTS

### Clinical and demographic data

**Figure 3:**
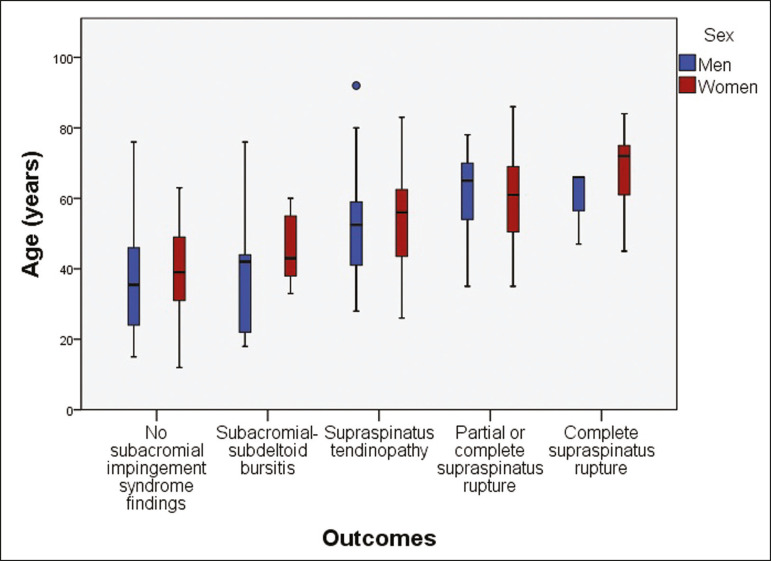
Boxplot demonstrating a tendency of increase in the severity of subacromial impingement syndrome with age and a higher prevalence of findings in women.

Three hundred and forty-six shoulders met the inclusion and exclusion criteria. One hundred and forty (40.5%) were men and 206 (59.5%) were women. The age ranged from 12 years to 92 years, with a mean, median, and standard deviation of 51 years, 52 years, and 16 years, respectively. Regarding the outcomes, we identified 81 (23.4%) shoulders without and 265 (76.6%) shoulders with signs of subacromial impingement syndrome. Among the patients with impingement, 19 (7.2%) had subacromial-subdeltoid bursitis, 137 (51.7%) had supraspinatus tendinopathy, 90 (34.0%) had partial supraspinatus rupture, and 19 (7.2%) had complete supraspinatus rupture. There was a higher prevalence of subacromial impingement syndrome findings in women and a tendency of increase in the severity of the impingement syndrome with advancing age (Figure 3).

### Univariate analysis

Statistically significant variables from the univariate analysis were sex (in all outcomes, except partial or complete supraspinatus tendon rupture), age (in all outcomes, except subacromial-subdeltoid bursitis), glenoacromial angle measured by observer A (in subacromial impingement syndrome, partial or complete supraspinatus tendon and complete supraspinatus tendon rupture outcomes), and the acromioclavicular angle measured by observer A (in partial or complete supraspinatus tendon rupture outcome-data summarized in Table 1).

**Table 1:** Univariate analysis results.

Outcome	Variable	*P*
Subacromial impingement syndrome	Sex	0.001 *
	Age	< 0.001 *
	Glenoacromial angle measured by observer A	0.032 *
	Glenoacromial angle measured by observer B	0.837
	Acromioclavicular angle measured by observer A	0.306
	Acromioclavicular angle measured by observer B	0.436
Subacromial-subdeltoid bursitis	Sex	0.049 *
	Age	0.091
	Glenoacromial angle measured by observer A	0.638
	Glenoacromial angle measured by observer B	0.722
	Acromioclavicular angle measured by observer A	0.685
	Acromioclavicular angle measured by observer B	0.271
Supraspinatus tendinopathy	Sex	0.005 *
	Age	< 0.001 *
	Glenoacromial angle measured by observer A	0.139
	Glenoacromial angle measured by observer B	0.939
	Acromioclavicular angle measured by observer A	0.810
	Acromioclavicular angle measured by observer B	0.804
Partial or complete supraspinatus tendon rupture	Sex	0.175
	Age	< 0.001 *
	Glenoacromial angle measured by observer A	0.012 *
	Glenoacromial angle measured by observer B	0.937
	Acromioclavicular angle measured by observer A	0.002 *
	Acromioclavicular angle measured by observer B	0.200
Complete supraspinatus tendon rupture	Sex	0.024 *
	Age	< 0.001 *
	Glenoacromial angle measured by observer A	0.001 *
	Glenoacromial angle measured by observer B	0.272
	Acromioclavicular angle measured by observer A	0.395
	Acromioclavicular angle measured by observer B	0.864

* Statistically significant variables (p < 0.05).

### Multivariate analysis

In the multivariate evaluation, a logistic regression was performed to ascertain the effects of sex, age, and glenoacromial and acromioclavicular angles on the likelihood that participants have subacromial impingement syndrome and supraspinatus tendon rupture (Table 2).

**Table 2:** Multivariate analysis results.

Outcome	Variable	OR	OR 95% CI	P
Subacromial impingement syndrome	Sex (male)	0.556	0.312–0.990	0.046 *
	Age	1.081	1.059–1.103	< 0.001 *
	Glenoacromial angle measured by observer A	1.029	1.016–1.043	< 0.001 *
Partial or complete supraspinatus tendon rupture	Sex (male)	0.810	0.436–1.507	0.507
	Age	1.073	1.049–1.095	< 0.001 *
	Glenoacromial angle measured by observer A	1.022	0.976–1.072	0.347
	Acromioclavicular angle measured by observer A	1.098	1.032–1.168	0.003 *
Complete supraspinatus tendon rupture	Sex (male)	0.426	0.115–1.580	0.202
	Age	1.084	1.044–1.125	< 0.001 *
	Glenoacromial angle measured by observer A	1.107	1.068–1.147	< 0.001 *

* Statistically significant variables (p < 0.05).

Male sex was a protective factor for subacromial impingement syndrome (odds ratio [OR] = 0.556; *p* = 0.046).

Increasing age and acromioclavicular angle measured by observer A were associated with a slight increase in the likelihood of supraspinatus tendon rupture: for an increase in age by each year or acromioclavicular angle by 1°, the OR increased by 1.073 and 1.098, respectively, for supraspinatus tendon rupture.

A decrease in glenoacromial angle measured by observer A also was associated with a slight increase in the likelihood of subacromial impingement syndrome (for each decrease in angle, the OR increased by 1.029) and complete supraspinatus rupture (for each decrease in angle, the OR increased by 1.107).

### Diagnostic performance analysis

**Figure 4:**
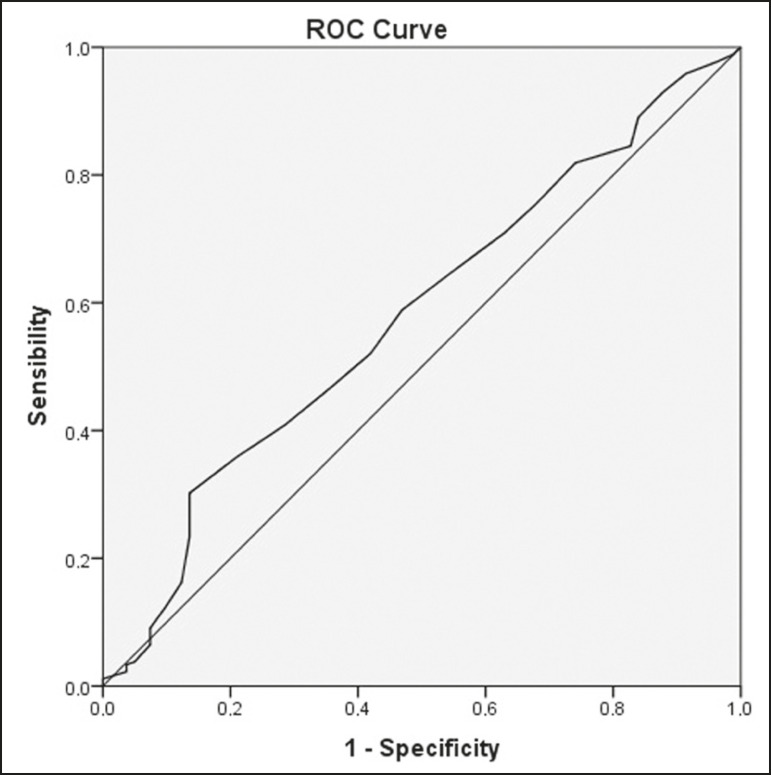
ROC curve plotted with glenoacromial angle measured by observer A for the “subacromial impingement syndrome” outcome.

**Figure 5:**
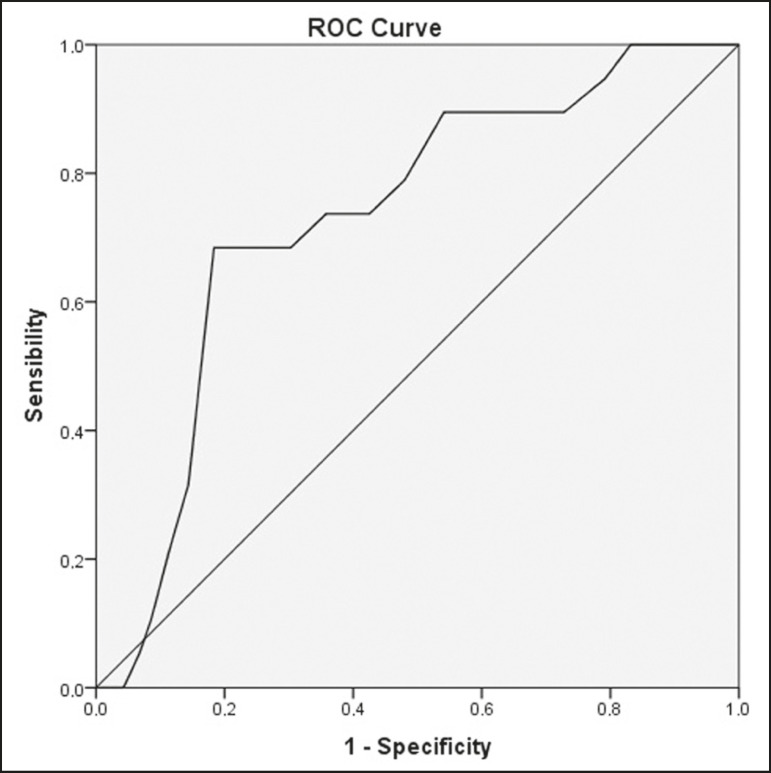
ROC curve plotted with glenoacromial angle mesured by observer A for the “complete supraspinatus tendon rupture” outcome.

The ROC curves plotted for the glenoacromial angle resulted in an AUC of 0.579 (95% CI: 0.508-0.649; *p* = 0.032) for the “subacromial impingement syndrome” outcome (Figure 4) and 0.731 (95% CI: 0.626-0.837; *p* = 0.001) for the “complete supraspinatus tendon rupture” outcome (Figure 5). The best cut-off angle determined by Yuden’s statistic was 72° (sensitivity, 30.2%; specificity, 86.4%; accuracy, 58.3%) for subacromial impingement syndrome and 69° (sensitivity, 68.4%; specificity, 81.7%; accuracy, 75.0%) for complete supraspinatus tendon rupture. For the glenoacromial angle, an OR of 2.1 (95% CI: 1.136-4.053) for subacromial impingement syndrome using the 72° cut-off value and an OR of 8.496 (95% CI 2.883-28.33) for complete supraspinatus tendon rupture using the 69° cut-off value were observed.

**Figure 6:**
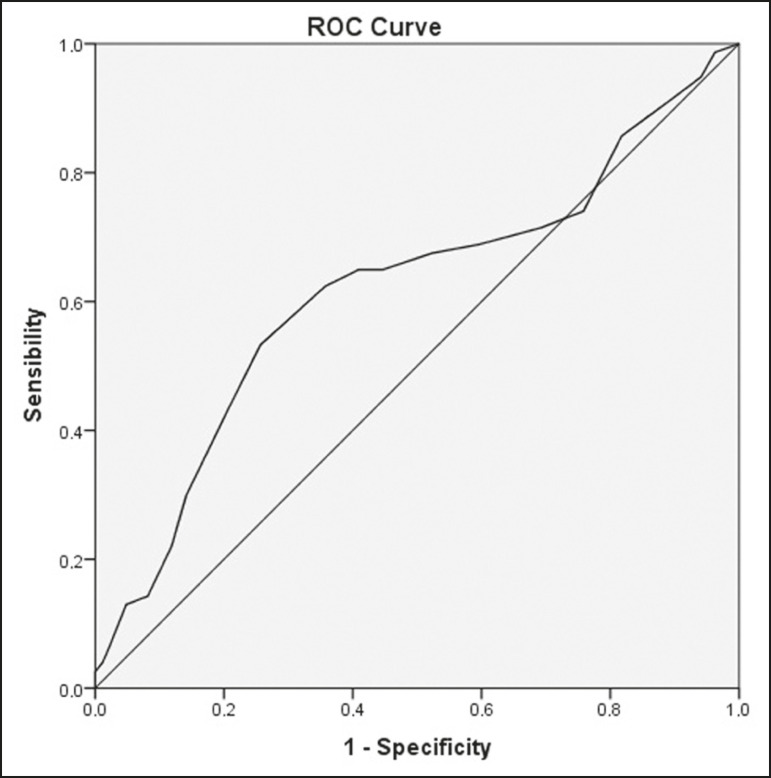
ROC curve plotted with acromioclavicular angle measured by observer A for the “supraspinatus tendon rupture” outcome.

The ROC curve (Figure 6) plotted with acromioclavicular angle measured by observer A for supraspinatus tendon rupture resulted in an AUC of 0.617 (95% CI: 0.539-0.694; *p* = 0.002). The best cut-off angle determined by Yuden’s statistic was 17° (sensitivity, 53.2%; specificity, 74.3%; accuracy, 63.8%). An OR of 3.288 (95% CI: 1.886-5.768) for partial or complete supraspinatus tendon rupture was observed using the 17° cut-off value of the acromioclavicular angle.

**Figure 7:**
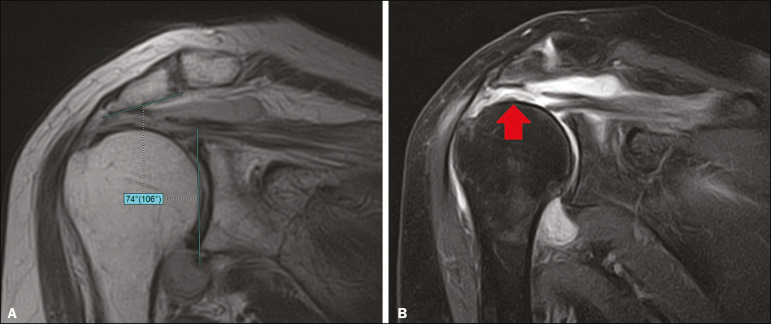
A 82 year-old man with signs of complete rupture of the supraspinatus tendon. **A**: Proton density-weighted image in the oblique coronal plane at the level of the acromioclavicular joint presenting a glenoacromial angle of 74°. **B**: T2-weighted image with fat saturation in the oblique coronal plane at the level of the supraspinatus tendon showing an interruption of the tendon fibers (arrow), characterizing complete rupture.

**Figure 8:**
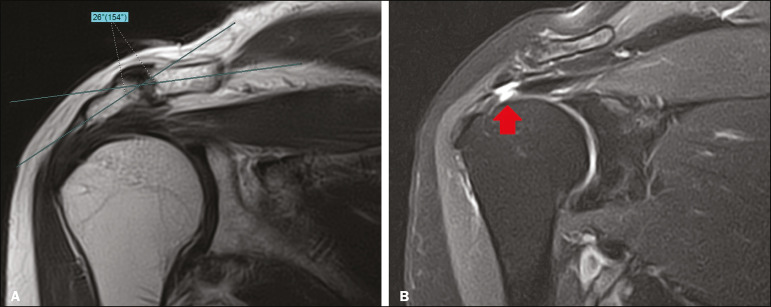
A 77 year-old woman with signs of a supraspinatus tendon transfixing rupture. **A**: Proton density-weighted image in the oblique coronal plane at the level of the acromioclavicular joint presenting an acromioclavicular angle of 26°. **B**: T2-weighted image with fat saturation in the oblique coronal plane at the supraspinatus tendon level depicting an interruption of some of the tendon fibers (arrow), characterizing rupture.

The measurement of glenoacromial and acromioclavicular angles is illustrated in Figures 7 and 8, respectively.

### Interobserver variability

A poor to moderate interobserver agreement was found between the glenoacromial and acromioclavicular angle measurements by observer A and observer B. The average ICC measure of the glenoacromial angle was 0.737 (95% CI: 0.676-0.787; *p* = 0.001) and that of the acromioclavicular angle was 0.507 (95% CI: 0.391-0.601; *p* = 0.001).

## DISCUSSION

We evaluated the effect of the acromial inferolateral tilt on subacromial impingement syndrome using the glenoacromial and acromioclavicular angles on shoulder MRI and the interobserver variability of these measurements. Reduction of the glenoacromial angle was an independent risk factor for subacromial impingement syndrome (*p* < 0.001) and complete supraspinatus tendon rupture (*p* < 0.001) according to observer A. Furthermore, an increase in the acromioclavicular angle was a risk factor for rupture (partial or complete) of the supraspinatus tendon according to observer A (*p* = 0.003). Among these three statistically significant relationships, we identified a lower interobserver variability of the glenoacromial angle (ICC = 0.737, i.e., moderate agreement) and a higher diagnostic performance of this parameter to assess the risk of complete supraspinatus tendon rupture (AUC = 0.731, i.e., fair performance).

The effect of the inferolateral acromial tilt on subacromial impingement syndrome has been poorly studied so far. To the best of our knowledge, only Banas et al.^(15)^, MacGillivray et al.^(19)^, Yao et al.^(6)^, Tétreault et al.^(23)^, Hanciau et al.^(24)^, and Balke et al.^(18)^ studied the relationship between the acromial inferolateral tilt angle and subacromial impingement syndrome. The methods of quantifying the inferolateral tilt and the results varied significantly among these authors.

Banas et al.^(15)^, Tétreault et al.^(23)^, Hanciau et al.^(24)^, and Balke et al.^(18)^ defined the acromial inferolateral tilt as the angle between the lower surface of the acromion and the glenoid face in the oblique coronal plane. Although all of these authors found significant relationships between the glenoacromial angle and the studied outcomes, the methodology varied significantly between them. The glenoacromial angle measurement in the series by Banas et al.^(15)^ and Tétreault et al.^(23)^ were performed on MR images, but the significant outcomes were different. Banas et al.^(15)^ identified a statistically significant correlation of the glenoacromial angle with the modified Zlatkin rotator cuff score (*p* < 0.0001) and the supraspinatus tendon grade (*p* < 0.0001) in 100 included shoulders. Tétreault et al.^(23)^ evaluated 94 shoulder MR images and found a statistically significant relationship between the glenoacromial angle and rotator cuff rupture relative to a control group without rotator cuff alteration (respective means 76 versus 86; *p* < 0.001).

The glenoacromial angle in the series by Hanciau et al.^(24)^ and Balke et al.^(18)^ was measured on shoulder radiographs (true anteroposterior view), and the dependent variable was defined clinically or by arthroscopy. Hanciau et al.^(14)^ included 55 shoulders in their work and found that 82.35% of the symptomatic patients (positive Neer test) had an angle of < 75°. Balke et al.^(18)^ included 150 shoulders and identified the following: 1) absence of a statistically significant relationship between glenoacromial angle and impingement symptoms (i.e. 84° versus 83°, respectively; *p* = 0.3); 2) statistically significant difference between patients with rotator cuff rupture evidenced by arthroscopy and without impingement symptoms (i.e. 77° versus 84°, respectively; *p* < 0.001); and 3) statistically significant difference between patients with rotator cuff rupture evidenced by arthroscopy and with impingement symptoms, but intact rotator cuff (i.e. 77° versus 83°, respectively; *p* < 0.001). The clinical definition of the outcome may have resulted from an inclusion of asymptomatic patients with rupture in the control group, resulting in a selection bias that may have compromised the results of the analyses.

Our results partially reproduced the findings of these authors probably because of differences in methods. Relationships of the glenoacromial angle of one of the researchers with subacromial impingement syndrome and complete supraspinatus tendon rupture were also evidenced; however, the best cut-off values of the present study were significantly different (72° for impingement syndrome and 69° for rupture), and a poor diagnostic performance of the glenoacromial angle for impingement syndrome and a fair diagnostic performance for complete supraspinatus rupture were observed.

Yao et al.^(6)^ and MacGillivray et al.^(19)^ evaluated the acromial inferolateral tilt by using other methods. In the study by Yao et al., 58 shoulder MR images were included, and the acromial inferolateral tilt accentuation was defined in two ways: subjectively and by an angle between the lower surface of the acromion and the superolateral glenoid extremity. These authors did not observe a statistically significant relationship between impingement and the subjective or quantitative assessment^(6)^. Therefore, because of the absence of statistical significance, this method was not included in the present study. MacGillivray et al. described the acromioclavicular angle and defined acromial inferolateral tilt accentuation as an angle superior to 10°. They included 132 shoulder MR images and identified 35 (27%) shoulders with an acromioclavicular angle greater than 10°, of which 85% showed signs of tendinopathy or rupture. Although it is unclear whether any hypothesis testing was applied, the authors concluded that the accentuation of the acromial inferolateral tilt could significantly impact the pathogenesis of impingement syndrome^(19)^.

The findings obtained in the present study only partially corroborate the results of these authors, possibly because of methodological differences. There was a statistically significant relationship between the acromioclavicular angle measured by one of the researchers and supraspinatus tendon rupture; however, there was a poor diagnostic performance.

Another finding consistent with literature was the interobserver variability of the inferolateral acromial tilt quantification. Yao et al.^(6)^ and Tétreault et al.^(23)^ also found poor to moderate interobserver agreement. The subjective characterization of the accentuation of the inferolateral acromial tilt may be influenced by the observer, as its report may be suggested by the presence of subacromial impingement syndrome. Therefore, in theory, a quantitative assessment of the inferolateral acromial tilt would be preferable; however, the measurement of the glenoacromial and acromioclavicular angles may be influenced by the structures characterized in the images. Not all examinations have an oblique coronal plane image that sufficiently depicts all the structures required for the angle measurements, and image scrolling may influence the measurements and lead to a higher interobserver variability.

Rotator cuff reconstruction may be accompanied by acromioplasty^(25)^. Evidence of higher risk for complete supraspinatus tendon rupture in patients with acromial inferolateral tilt accentuation may influence the orthopedists to associate acromioplasty to the reconstruction. Therefore, the measurement of the glenoacromial angle in cases of complete rupture of the supraspinatus tendon may influence the surgical approach.

The main limitations of our work were the retrospective design and the dependence on MRI findings to define the outcomes. A prospective design would be preferable with the inclusion of other variables, such as dominance, symptoms, and arthroscopy findings. The major strengths of this study are the inclusion of a large sample, the comparison of different measurements, and the assessment of diagnostic performance and interobserver agreement of each measurement method. The inclusion of a larger sample in this study than in previous studies implies that the results are more accurate with smaller confidence intervals. The comparison of diagnostic performance and interobserver variability of different acromial inferolateral tilt quantification methods allowed us to identify the best parameter among those described.

## CONCLUSION

Inferolateral acromial tilt may have some impact on subacromial impingement syndrome and supraspinatus tendon rupture. Among all the variables studied, the most appropriate parameter for acromial inferolateral tilt quantification found in this series was the glenoacromial angle, considering the diagnostic performance and interobserver agreement; however, it showed a moderate interobserver agreement and a fair performance to assess the risk of complete supraspinatus tendon rupture.
